# Anatomical Variants of the Renal Veins and Their Relationship with Morphofunctional Alterations of the Kidney: A Systematic Review and Meta-Analysis of Prevalence

**DOI:** 10.3390/jcm13133689

**Published:** 2024-06-25

**Authors:** Juan Jose Valenzuela Fuenzalida, Karla Vera-Tapia, Camila Urzúa-Márquez, Javiera Yáñez-Castillo, Martín Trujillo-Riveros, Zmilovan Koscina, Mathias Orellana-Donoso, Pablo Nova-Baeza, Alejandra Suazo-Santibañez, Juan Sanchis-Gimeno, Alejandro Bruna-Mejias, Héctor Gutiérrez Espinoza

**Affiliations:** 1 Departamento de Morfología, Facultad de Medicina, Universidad Andrés Bello, Santiago 8370146, Chilekarlavera@uandresbello.edu (K.V.-T.); javierayanez@uandresbello.edu (J.Y.-C.); mtrujillo@uandresbello.edu (M.T.-R.); zkoscina@uandresbello.edu (Z.K.); mathias.orellana@unab.cl (M.O.-D.); pablo.nova@usach.cl (P.N.-B.); 2 Departamento de Ciencias Química y Biológicas, Facultad de Ciencias de la Salud, Universidad Bernardo O’Higgins, Santiago 8370993, Chile; 3 Escuela de Medicina, Universidad Finis Terrae, Santiago 7501015, Chile; 4 Faculty of Health and Social Sciences, Universidad de Las Américas, Santiago 8370040, Chile; alejsuazo@uandresbello.edu; 5 GIAVAL Research Group, Department of Anatomy and Human Embryology, Faculty of Medicine, University of Valencia, 46001 Valencia, Spain; 6 Departamento de Ciencias y Geografía, Facultad de Ciencias Naturales y Exactas, Universidad de Playa Ancha, Valparaíso 2360072, Chile; 7 One Health Research Group, Universidad de Las Américas, Quito 170124, Ecuador

**Keywords:** renal vein, kidney failure, variations renal veins, vascularization kidney, variation anatomical, kidney surgery, kidney transplant

## Abstract

**Background:** Variations in renal veins are quite common, and most people do not experience issues due to them. However, these variations are important for healthcare professionals, especially in surgical procedures and imaging studies, as precise knowledge of vascular anatomy is essential to avoid complications during medical interventions. The purpose of this study was to expose the frequency of anatomical variations in the renal vein (RV) and detail their relationship with the retroperitoneal and renal regions. **Methods:** A systematic search was conducted in the Medline, Scopus, Web of Science, Google Scholar, CINAHL, and LILACS databases from their inception until January 2024. Two authors independently carried out the search, study selection, and data extraction and assessed methodological quality using a quality assurance tool for anatomical studies (AQUA). Ultimately, consolidated prevalence was estimated using a random effects model. **Results:** In total, 91 studies meeting the eligibility criteria were identified. This study included 91 investigations with a total of 46,664 subjects; the meta-analysis encompassed 64 studies. The overall prevalence of multiple renal veins was 5%, with a confidence interval (CI) of 4% to 5%. The prevalence of the renal vein trajectory was 5%, with a CI of 4% to 5%. The prevalence of renal vein branching was 3%, with a CI of 0% to 6%. Lastly, the prevalence of unusual renal vein origin was 2%, with a CI of 1% to 4%. **Conclusions:** The analysis of these variants is crucial for both surgical clinical management and the treatment of patients with renal transplant and hemodialysis.

## 1. Introduction

In their usual route, the renal veins (RVs) form in the renal hilum. Along this route, the right renal vein (RRV) receives tributaries, while the left renal vein (LRV) receives the left adrenal vein and the left gonadal vein, ending its journey in the inferior vena cava (IVC) at the level of the L1 vertebra. However, they do not present this way in all individuals, since variants may arise in embryonic development [1,2]. Particularly, when studying RV anatomy, any greater complexity in the RV, due to its relationship with the abdominal aorta (AA) and the superior mesenteric artery (SMA), has its beginning in the embryonic development of these vessels. If anomalies occur in the embryogenesis of this vein, it can surround the AA or the discourse posterior to it. These phenomena are known as the circumaortic renal vein or retroaortic renal vein, respectively. Another important RV variation involves supernumerary veins, also known as multiple RV; instead of one venous trunk, up to four can be found. These variations are more frequently associated with the RRV. Finally, another variation occurs in the accessory vessels that contribute to the RV, including the posterior tributary vein, which connects the posterior course with the renal pelvis [3,4,5,6].

These RV variations have been widely described in meta-analyses of case studies and cadaveric dissections, although the statistics and analysis of their incidences are hardly discussed in the literature. Several studies highlight the importance of these variations in the clinical context. Although they generally do not present symptoms, associations have been described, such as RV hypertension syndrome, in which renal venous hypertension causes venous flow to be directed retrogradely towards the renal parenchyma, generating ruptures of veins in the collecting system. Another syndrome studied in association with these variations is posterior nutcracker syndrome, which presents with macroscopic hematuria and/or associated proteinuria due to compression of the RV. These syndromes have been associated with both variations of LRV described above. Understanding these variations and their incidences can prevent unfavorable results or poor intraoperative practices. The importance of the different renal patterns in renal transplantation and radical nephrectomy cannot be underestimated. Knowledge of the architecture of the renal vessels and a study beforehand to clarify the presence of these variations can be essential to the success of these procedures, especially with the great radiological advances in recent years [7,8,9].

The objective of this review was to know the characteristics and prevalence of the anatomical variants of RV and their relationship with renal pathologies and the importance of knowledge of this anatomical variant in surgeries.

## 2. Methodology

### 2.1. Protocol and Registration

To carry out this meta-analysis, we were guided by the Prisma statement. The registration number in the Systematic Reviews Registry (PROSPERO) is CRD42022224066.

### 2.2. Electronic Search

In order to have the best studies that fit our research question, we searched the following databases during the months of October and November: MEDLINE (via PubMed), Google Scholar, Web of Science (WOS), Cumulative Index to Nursing and Allied Health Literature (CINAHL), Latin American and the Caribbean Literature in Health Sciences (LILACS), and Scopus from its inception until November 2023. Our search strategy included a combination of the following terms: “renal vein” (Mesh), “renal failure” (not Mesh), “renal vein variations” (not Mesh), “vascularization kidney” (not Mesh), “anatomical variation” (not Mesh), “kidney surgery” (Mesh), and “kidney transplant” (Mesh), using the Boolean connectors AND, OR, and NOT.

### 2.3. Eligibility Criteria

As eligibility criteria, the studies that were included considered the presence of RV variants and their association with some clinical conditions. They were considered eligible for inclusion if the following criteria were met: (1) sample: dissections or images with the presence of the RV variation; (2) results: prevalence of subjects who presented RV variants and their correlation with pathologies of the retroperitoneal region; (3) studies: this systematic review included research articles, retrospective and prospective observational types, published in English in peer-reviewed journals, and indexed in the reviewed databases.

As exclusion criteria we used the following to eliminate from our selection: (1) sample: studies carried out in animals; (2) studies that analyzed variants of the venous system outside the renal region or its drainage area or tract; (3) letters to the editor or comments.

### 2.4. Study Selection

In order to make a thorough selection of the studies, we three authors analyzed the material independently. In the first instance, two authors (KV and MT) examined the titles and abstracts of the references recovered from the database searches. For the selected studies, the full text of the references that any of the authors considered potentially relevant was obtained. A third reviewer (PN) was involved if a consensus could not be reached. For this purpose, we also performed the agreement test between authors, the kappa test, to analyze reliability and the risk of bias between observers, which in this case gave 0.70, which is interpreted as a good agreement.

### 2.5. Data Collection Process

Two authors (MO and KV) independently extracted data on the outcomes of each study. The following data were extracted from the included studies: (a) authors and year of publication, (b) country, (c) type of study and number of participants, (d) sample characteristics and prevalence, (e) reported statistical values, (f) region geography of the study, (g) sex of the sample, and (h) laterality of the presence of the variant (right, left, and bilateral).

### 2.6. Assessment of the Methodological Quality of the Included Studies

To evaluate the bias of the included studies, we used the verification table for anatomical studies (AQUA) proposed by the International Working Group on Evidence-Based Anatomy (IEBA) [10]. Two reviewers (JJV and JM) independently analyzed the 5 domains proposed by the AQUA tool, then reached a consensus and constructed the table and the bias graph.

### 2.7. Publication Bias

Through JAMOVI, we made funnel plots. For publication bias, we have the funnel plot graph, where theoretically the data that most affect this criterion are the statistical significance of the primary article and its sample; this graph crosses the sample measurement against the exposure association or confidence interval transformed into standard error against the sample size.

### 2.8. Statistical Methods

For the statistical analysis, we used the JAMOVI technological tool Version 4.0 2022 (R Core Team, 2021) [11]. Where we included the data in a binary way and continuously to obtain the proportion of the data which we expressed in prevalence, the statistical model used was the DerSimonian–Laird with a Freeman–Tukey double-arcsine transformation to combine the summarized data. Additionally, a random effects model was used because the VD prevalence data were very heterogeneous. The degree of heterogeneity among the included studies was assessed using the chi^2^ test and the heterogeneity statistic (I^2^). Finally, with the JAMOVI tool, we analyzed a funnel plot graph where the magnitude of the measured effect is represented, which is graphed in a funnel plot [10].

## 3. Results

### 3.1. Included Articles

**Figure 1:**
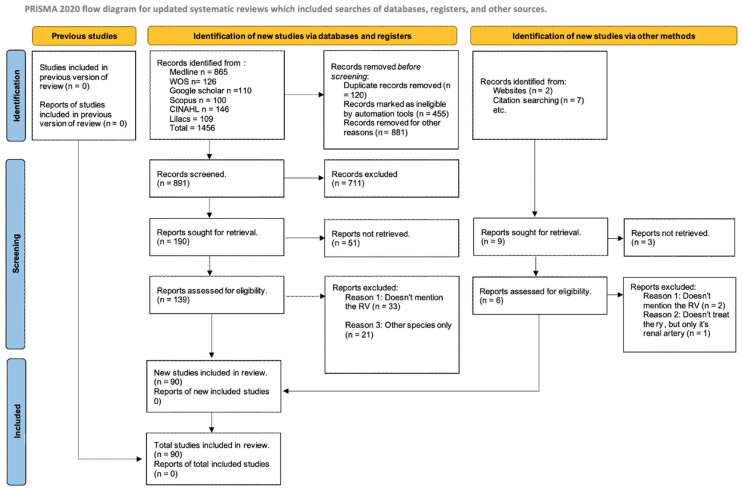
Flow search diagram [12].

The researchers identified a total of 1456 articles in various databases that met the established criteria and search terms. Titles and/or abstracts of the articles in the consulted databases were filtered, primarily using duplicate elimination as the initial criterion. Subsequently, 180 full-text articles were analyzed to determine their eligibility in this meta-analysis and systematic review. A total of 148 studies were excluded due to discrepancies in primary and secondary outcomes concerning this review and not meeting the criteria for corresponding data extraction. As a result, 90 articles (*n* = 46,664) were included for analysis, encompassing patients, images, and cadavers (Figure 1).

### 3.2. Characteristics of the Studies and the Study Population

**Figure 2:**
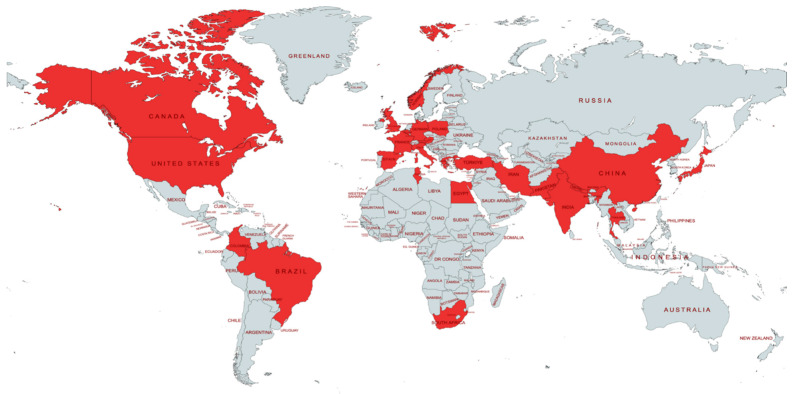
Geographic distribution of studies and subjects included in this review.

The samples analyzed in the reviewed studies came from all continents except Oceania. In Europe, 36 studies were conducted, representing 40% of the total. The cumulative number of patients in these studies was 33,790, consolidating 72.41% of the reviewed samples. A total of 18 studies (20%) were carried out in Asia, with a total of 3368 patients, representing 7.22% of the analyzed samples. In North America, 21 studies were conducted (23.3%), with a cumulative number of 7048 patients, representing 15.1% of the samples. South America had nine studies (10%) with a total of 969 patients, accounting for 2.07% of the samples in our analysis (Table 1 and Figure 2). Finally, in Africa, six studies were conducted (6.6%), with a cumulative total of 1489 patients, representing 3.19% of the sample size in our analysis.

Regarding the focus of the studies, 42 analyzed the renal vein bilaterally, while 4 focused only on the right side and 44 only on the left side. In addition, of the patients included in the reviewed studies, 31.91% were male, 25.63% were female, and 41.19% chose not to specify their gender (Table 1).

### 3.3. Description of Variants

**Figure 3:**
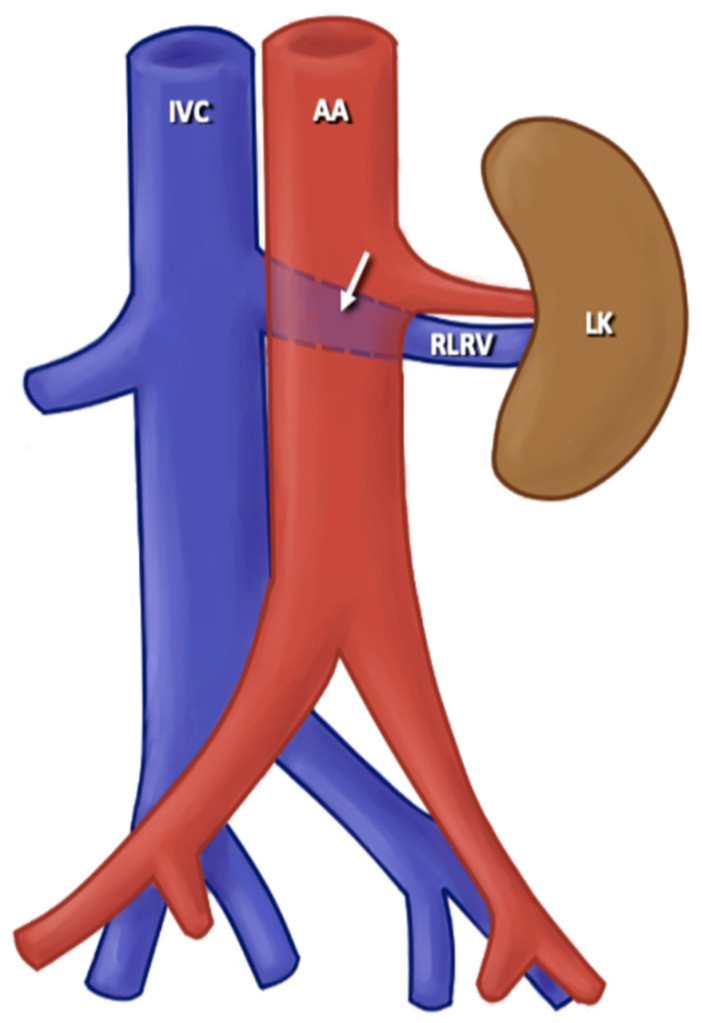
Retroaortic left renal vein. AA: abdominal aortic; ICV: inferior caval vein; CLRV: circumaortic left renal vein; LK: left kidney.

**Figure 4:**
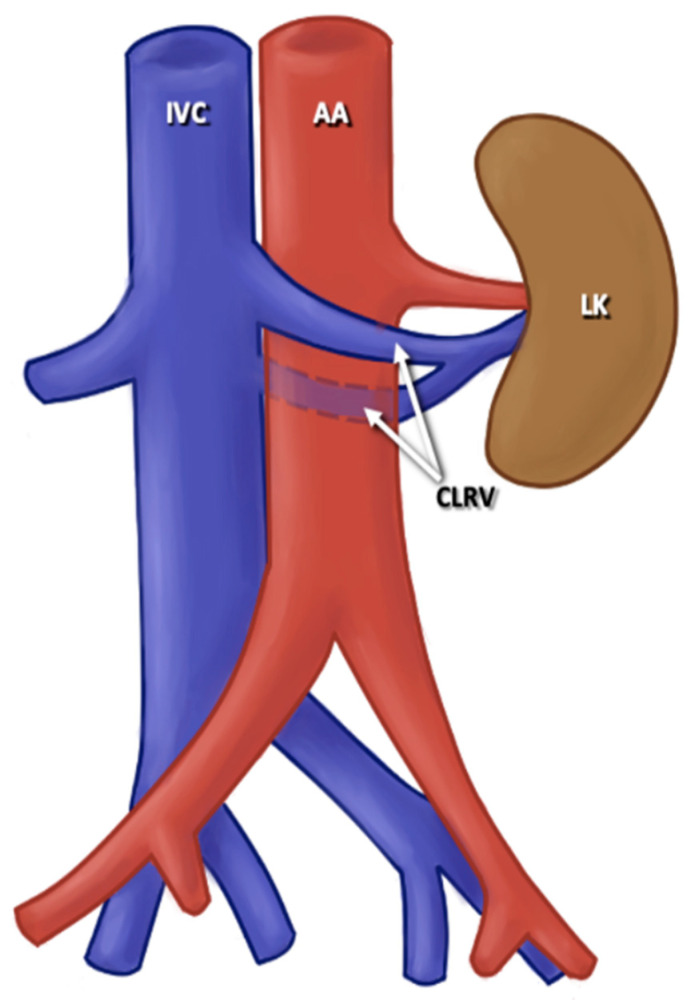
Circumaortic left renal vein. AA: abdominal aortic; ICV: inferior caval vein; CLRV circumaortic left renal vein; LK: left kidney.

**Figure 5:**
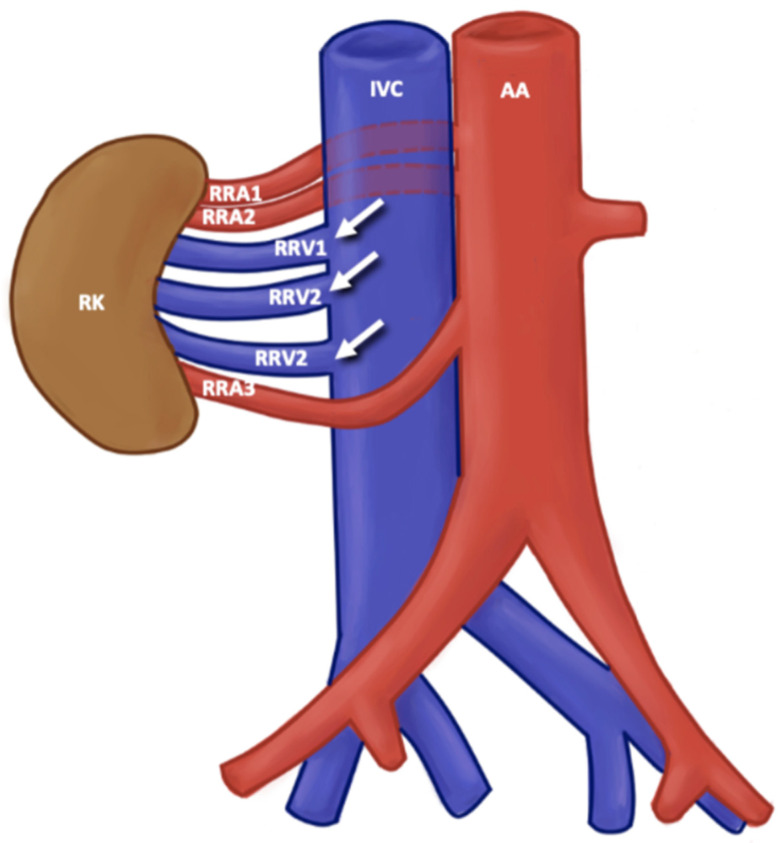
Multiple renal veins. AA: abdominal aortic; ICV: inferior caval vein; RK: right kidney; RRA3 right renal arteries; RRV1, RRV2, RRV3: right renal vein.

Among the RV variants found in the literature that we analyzed in this prevalence study, variations were found at the level of origin of the RV and the trajectory of the RV; additionally, some cases included multiple RV and variations in the ramifications of the RV. For the variants in the origin of the RV, a variant of origin of LRV was considered any situation in which the RV, both unilaterally and bilaterally, arose from a level lower than L2-L3 from the IVC; the RV arose from a different site to the IVC; the drainage occurred at the level of the lateral aspect of the IVC; or a late venous confluence was present where both the origin of the RV and its path towards the renal hilum were affected. For the RV course variants, the normal course of the RV was considered in which the RV crossed the anterior part of the AA to drain into the IVC. The variants observed in the literature with the highest prevalence were the retroaortic and circumaortic paths of RV (Figure 3 and Figure 4). A retroaortic RV path is any path in which the RV crosses the posterior part of the AA, finally draining into the IVC; a circumaortic course is one in which the RV forms a circle around the AA and drains into the IVC. For the multiple RV variant, all RVs with a single vascular trunk were considered normal. The ones with double, triple, and quadruple trunk of the RV were considered multiple RV variants, either unilaterally or bilaterally. Finally, all cases in which the RV had one or more accessory branches and the latter ending up draining into the IVC were considered the RV branching variant (Figure 5).

### 3.4. Prevalence

**Figure 6:**
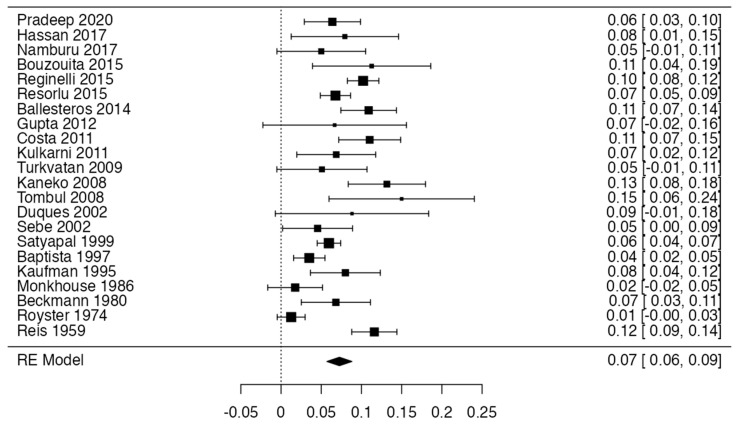
Multiple RV forest plot.

**Figure 7:**
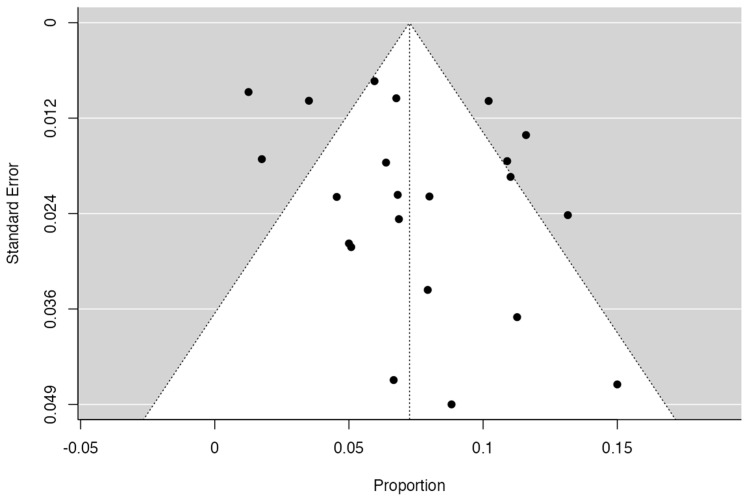
Multiple RV funnel plot.

**Figure 8:**
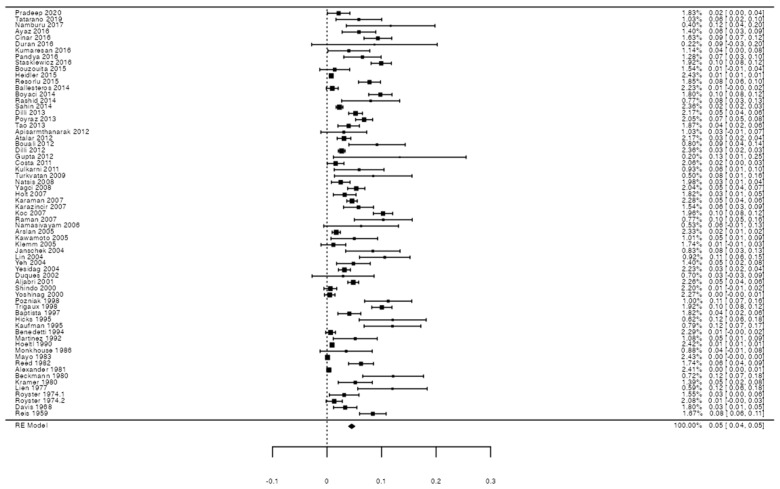
RV course forest plot.

**Figure 9:**
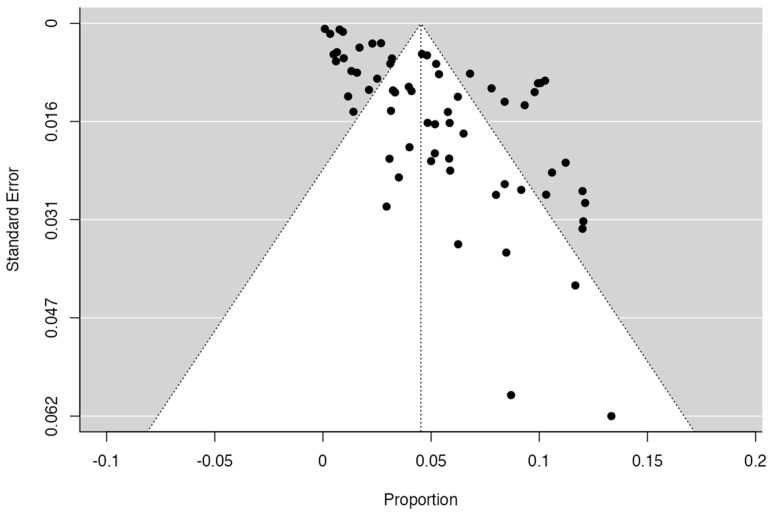
RV course funnel plot.

**Figure 10:**
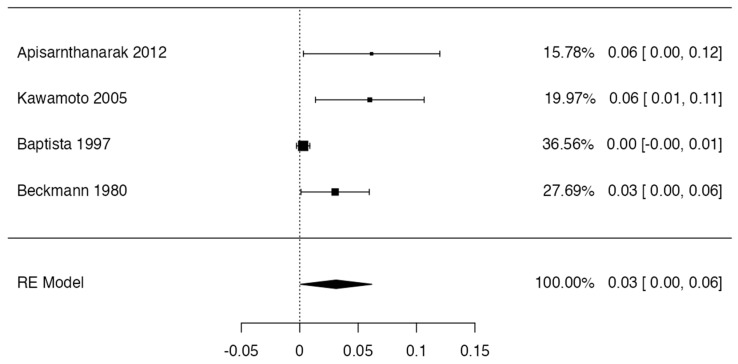
Forest plot branches RV.

**Figure 11:**
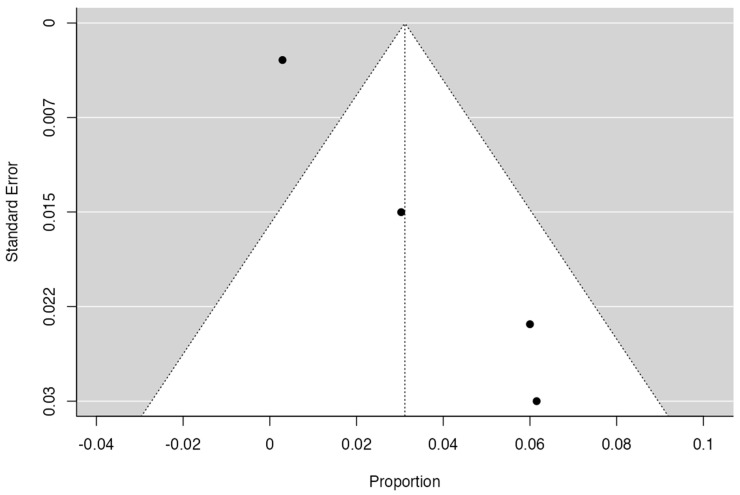
Funnel plot branches RV.

**Figure 12:**
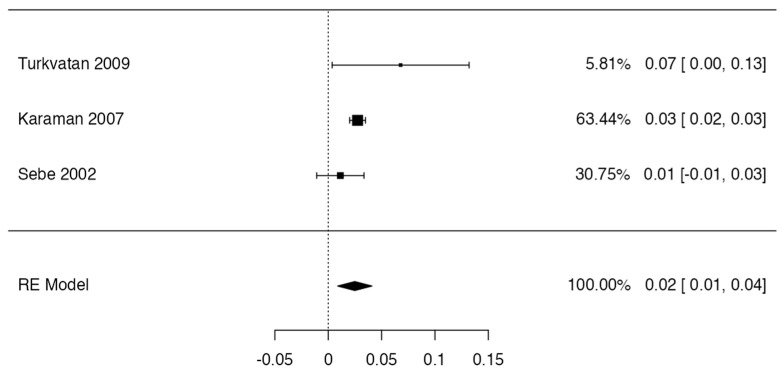
Forest plot unusual origin of RV.

**Figure 13:**
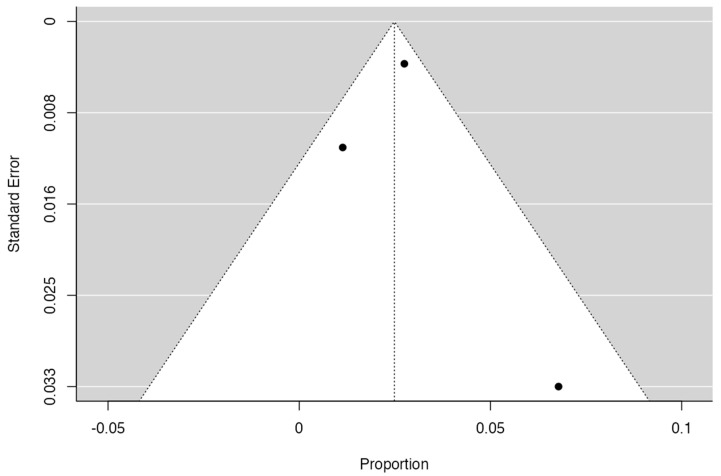
Funnel plot unusual origin of RV.

To calculate the prevalence of RV variants in the studies included in this review (Table 2), four proportion forest plots were made. For the multiple RV variant, a forest diagram was made with 22 studies (Figure 6) [15,20,21,28,30,31,32,33,49,50,53,54,57,72,77,80,82,83,86,90,94,97]. For this first sample, the funnel plot graph showed an important asymmetry which presented a *p* value of 0.412, which is directly related to this asymmetry (Figure 7). The diagram showed that the prevalence of multiple RV was 7%, with a confidence interval of 6% to 9%. For the RV course variant, 64 studies were included (Figure 8) [3,7,15,18,21,22,23,24,25,26,27,29,31,33,34,37,38,39,41,42,43,44,45,46,49,50,53,56,57,58,59,60,61,62,63,64,65,66,67,68,69,70,72,73,74,75,76,78,79,81,82,84,85,86,87,88,89,90,91,92,94,95,97,98]. For this second sample, the funnel plot graph showed an important asymmetry which presented a *p* value of 0.560, which is directly related to this asymmetry (Figure 9, and the prevalence of the RV course variant was 5%, with a confidence interval of 4% to 5%. The RV branching variant forest plot included four (Figure 10) [43,66,80,90]. For this third sample, the funnel plot graph showed an important asymmetry which presents a *p* value of 0.162, which is directly related to this asymmetry (Figure 11). The prevalence of RV ramifications was 3%, with a confidence interval of 0% to 6%. Finally, for the unusual origin of RV, three studies were analyzed, and the prevalence was 2%, with a confidence interval of 1% to 4% (Figure 12) [53,60,73]. For this fourth sample, the funnel plot graph showed an important asymmetry which presented a *p* value of 0.382, which is directly related to this asymmetry (Figure 13). The pooled prevalence of studies independently in prospective studies (49 studies) and retrospective studies (15 studies) was calculated. The prevalence in prospective studies was *p* = 0.413 (95% CI 0.323–0.504), and in retrospective studies, it was *p* = 0.278 (95% CI 0.03–0.526). It is noted that the difference was not significant. Additionally, it is important to highlight that heterogeneity was very high in both groups (I2 91.63% in prospective studies and 92.62% in retrospective studies). Furthermore, it is worth noting that in both groups, there was publication bias based on the asymmetry of the funnel plot.

### 3.5. Risk of Bias of Included Articles

**Figure 14:**
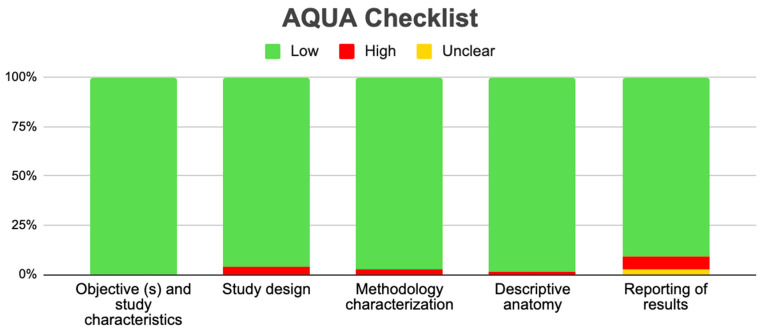
Graphic of AQUA checklist for included studies.

A total of 79 articles were evaluated with the AQUA checklist to analyze the risk of bias in five domains (Figure 10). For the first domain, which covers the description of the objectives and characteristics of the study, all studies presented a low risk of bias. The second domain is the correct reporting of the study design. A total of 76 studies presented a low risk of bias in this domain, and 3 presented a high risk since they did not clearly report the design of their studies [18,75,94]. For the third domain, which analyzes the study’s methodological characteristics, 77 studies presented a low risk of bias, while 2 presented a high risk since their methodology was unclear [32,70]. The fourth domain is the correct description of anatomy. A total of 78 studies presented a low risk of bias in this domain, while only 1 study presented a higher risk since it did not include an anatomical description of the variant but instead merely named it [88]. In the final domain, which involves reporting results, 72 studies presented a low risk of bias, 2 presented their results unclearly, and 5 studies presented a high risk of bias since their results were presented diffusely in tables or in discussion sections [18,57,75,88,94] (Figure 14).

For the analysis of studies with case report methodology, the JBI tool was used to assess the risk of bias. A total of 12 studies were analyzed within the eight domains of this bias tool [13,14,16,19,35,40,48,52,55,71,93,99]. The majority presented a low risk of bias in domains 1 to 6. However, in domain 7, which focuses on adverse events (harms) or unanticipated events, seven studies presented a high risk of bias [13,14,16,19,52,71,99]. Domain 8 analyzes whether the case report provides takeaway lessons. Seven studies presented a high risk of bias since they did not comply with what was proposed in this domain (Table 3 and Table 4) [13,14,16,40,52,55,71].

### 3.6. Clinical Considerations

Among the 90 studies analyzed in this review, 59 demonstrated some clinical correlation to the various anatomical variations of RV. For the most part, these variations are clinically silent [29,33,39,47,67,77,83]; however, when they produce symptoms, we can observe syndromes such as the “nutcracker syndrome” [2,22,24,29,41,65,100], which corresponds to a compression of the LRV in its retroaortic variation caused by the SMA (superior mesenteric artery) and the AA (abdominal aorta). This syndrome is rare and classically presents with proteinuria and hematuria; therefore, it is diagnosed through laboratory tests, such as urinalysis [25,29,31,34,39,42,58,65,83]. It can also have significant complications, such as dilation of the gonadal vein, generating varicocele in men [2,22,23,25,34,42,61,65], and pelvic congestion syndrome in women [29,39,58,83]. Varicocele is the dilation of the veins within the scrotum. It is usually asymptomatic but can cause a decrease in sperm production and quality, which may eventually lead to infertility. On the other hand, pelvic congestion syndrome in women is the accumulation of venous blood in the pelvis. This is a common cause of chronic pelvic pain in women and causes the appearance of varicose veins in the vulva, vagina, or thigh [34].

Preoperative knowledge of each of these anatomical variations is of utmost importance, since they can influence the viability of the procedure [77]. Understanding them helps facilitate the procedure’s safe performance [29,45,54] and reduce complications during and after retroperitoneal interventions, which include kidney transplantation, AA aneurysm surgery, gonadal surgery, lymphadenectomy, and nephrectomy [6,23,24,35,46,48,49,65,70,72,78,79,95]. The most prominent compilation is hemorrhage [28,32,34,43,74,80,89]. On the other hand, ignorance of these variables can compromise or complicate surgery [30,31,101] and even cause injury to some of these vessels [26,33,83,88]. Various types of imaging, such as computed axial tomography (CAT) angiography [33,63,78], abdominal computed tomography (CT) with contrast [44,45,46,52,88], and multidetector computed tomography (MDCT) [24,31,50,57,63,64,93,102], have been recommended to study the different anatomical variations of RV.

## 4. Discussion

This systematic review and meta-analysis aimed to report the anatomo-clinical characteristics and prevalence of RV variants and their association with pathologies of the kidney or surrounding structures. The main finding of our review was the correlation between the prevalence of RV variants and different surgeries of the renal region, as well as hemodialysis.

As we observed in this review, variants of RV can be of more than one type, including variants in the origin of RV or journey and entry to the IVC; increased numbers of RV, known as multiple RV, can also occur. Yi et al. (2012) [35] also analyzed RV variants. Only 27 studies were included, in contrast to the present study, which included 90 studies overall and 63 for the meta-analysis of RV journey prevalence. Furthermore, we believe that the prevalence of RV variants is overestimated in their review. They present very high values and define them as common variabilities. Our detailed study shows low prevalence in our different forest plots, suggesting that their data may have been calculated with values from primary studies that only looked for the variant.

The last manuscript associated with the variants of the RV was published in 2019, so this review updates the topic of RV over the past 5 years. In relation to the latter, we make a detailed review of the anatomy of the different variants of the renal vein, adding that we make a clinical correlation, which is why, apart from the years of the last publication on the RV variants, we approached the variant through translational anatomy and providing strong support between the anatomy and the clinical correlations. Hostiuc et al.’s (2019) [103] review does not detail the anatomical characteristics of each RV; in our study, we detailed the variants by subgroup. Their review included 105 studies with an accurate meta-analysis; it differs from our study in that they did not detail the clinical correlations of these. Furthermore, we provided a detailed anatomical description of each variant to provide clinical support for the study of translational anatomy of RV.

There was no indication in the included studies that RV variants had any type of relationship to the sex of the subjects. Similarly, there was no type of indication that RV variants are associated with any specific ethnicity or race; however, to further support this hypothesis, we suggest that more interracial studies should be carried out. With respect to laterality, there was also no type of indication in the studies that variants were associated with the left side or with the right side in specific ethnicities. Finally, age was a value that we did not consider in our study since variants are congenital and thus unrelated to the age of the subjects.

We grouped the variants as RV course variant, multiple RV variant, unusual origin of RV, and variant of RV ramifications. Studies that reported the RV course variant were more commonly found; this is associated with a retroaortic and anteroaortic passage, generating a kind of circumduction on the RV. We did not consider primary studies that showed low prevalences, because if we included all the studies, the results could have been overestimated. We believe that when the prevalence of the variants was high, it is because the sample was intentional and not random; this alters the data from the prevalence meta-analysis, so we decided to not include these results. We generated four prevalence forest plots and found a prevalence of 8% for multiple RV, a prevalence of 5% for course variants, a prevalence of 5% for RV ramifications variants, and a prevalence of 2% for unusual origin of the RV. Finally, we analyzed the publication bias through a funnel plot for each of the prevalence measurements, and we detected a high level of publication bias among some studies, which is why the data must be interpreted with caution.

The heterogeneity of the studies was between 80 and 97%, which is high and could over- or underestimate the reported results. Thus, they should be taken with caution, and we recommend further studies. The AQUA tool was used to assess the bias of the included studies. The results show a low risk of bias in the five domains in all the included studies; therefore the data were included with greater security for the analysis. The case reports presented greater bias in the analysis of results, so we only considered those that presented variants that were underrepresented in the literature or reported some important clinical correlation that supported their inclusion. Finally, while the clinical considerations reported in this study were varied, we focused above all on the intrasurgical care of the abdominal region and the retroperitoneal region, since these variants are often silent and their description or discovery is associated with routine examinations or pathologies of surrounding structures. The only syndrome reported with symptomatology is “nutcracker syndrome”, which typically presents signs such as hematuria and must be diagnosed with laboratory tests. This syndrome is very rare in the literature; unfortunately, no article presented a clear prevalence, but we estimate due to the amount of information on the subject that it is less than 1%.

In kidney transplantation, dilemmas can arise due to the positioning of the RV. In the presence of these variants, the veins have acquired an arrangement in the abdominal region, occupying uncommon regions. Patients are often asymptomatic, so many surgeons choose to maintain the arrangements of these variants in transplant surgery [104,105,106]. Finally, an equally important clinical correlation is the presence of RV variation before hemodialysis, which is associated with greater complexity in performing the catheterization, because the arrangement of the RV and the surrounding structures may be different. It has also been reported that this could increase the probability of clots or thrombi; a thorough analysis of the region can prevent these complex conditions.

## 5. Limitations

This review was limited by the publication and authorship bias of the included studies. First, studies with different results that were in the nonindexed literature in the selected databases may have been excluded. Second, there could be limitations in the sensitivity and specificity of the searches. Finally, the authors personally selected articles. All of this increases the probability of excluding potential cases from countries outside of Asia and North America that are not being reported in the scientific community.

## 6. Conclusions

The presence of RV variants has been widely described in the literature. Our results show that the variants of the renal vein can be multiple and that mainly, the lack of knowledge of these could cause iatrogenic injuries during surgeries of structures surrounding the kidney. Regarding patients who receive a kidney transplant and present the RV variant, the surgeon must know how this variant could make work more difficult with these patients; however, prior knowledge could help the surgery to be planned with all these considerations, and these changes could improve the probability of surgical success in these patients. We also believe that more studies that explain how this variant behaves and the symptoms associated with the variant could be necessary.

## Figures and Tables

**Table 1:** Characteristics of included studies.

Author and Year	Number of Patients	Incidence and Characteristics	Statistical Value	Geographic Region	Sex	Laterality
Pedrao, 2023 [13]	Observational N = 1	Double RRV.	Not mentioned.	Brazil	1 male	Right
Silva, 2021 [14]	Observational N = 1	Multiple retroaortic LRV.	Not mentioned.	Brazil	1 male	Left
Pradeep, 2020 [15]	Observational N = 188	Double RRV (12 cases—6.3%). Retroaortic LRV (4 cases—2.1%).	Not mentioned.	Nepal	Not specified	Bilateral
Salimy et al., 2020 [16]	Observational N = 1	Double RRV. Right kidney with double collector system.	Not mentioned.	USA	1 male	Right
Fontana, 2018 [17]	Observational N = 1	Duplication of circumaortic RV.	Not mentioned.	Italy	1 male	Left
Tatarano et al., 2019 [18]	Observational N = 120	Circumaortic LRV (6 cases). Retroaortic LRV (1 case).	There was no significant incidence between donors with or without abnormalities on their LRV.	Japan	Not specified	Left
Dunnwald, 2019 [19]	Observational N = 1	Triple RRV (1 case).	Not mentioned.	USA	1 male	Bilateral
Shaheen, 2018 [6]	Observational N = 50	Double RRV (28 cases—56%), triple (13 cases—26%), quadruple (5 cases—10%).	Not mentioned.	Pakistan	50 males	Bilateral
Hassan, 2017 [20]	Observational N = 63	Double RV (2 cases—3%), triple (2 cases—3%), quadruple (1 case—2%).	No significant differences between cadavers with or without variations in renal vasculature and age of death (*p* = 0.67) or gender (*p* = 0.71).	Egypt	32 males 31 females	Bilateral
Nambur, 2017 [21]	Observational N = 60	Circumaortic LRV (2 cases—3.3%). Retroaortic LRV (5 cases—8.3%). Double RRV (3 cases—5%). Right gonadal vein draining into RRV (1 case—0.3%).	Not mentioned.	India	Not specified	Bilateral
Ayaz, 2016 [22]	Observational N = 222	Circumaortic LRV (7 cases—3.15%). Retroaortic LRV (6 cases—2.7%).	Not mentioned.	Turkey	116 males 106 females	Left
Çınar, 2016 [23]	Observational N = 504	Circumaortic LRV (26 cases—5.2%). Retroaortic LRV (21 cases—4.2%). RRV: double (97 cases—19.2%), triple (11 cases—2.2%), quadruple (1 case—0.2%).	No associations were found between sex and the presence of RA or RV variations (*p* = 0.630 and 0.650, respectively).	Turkey	317 males 187 females	Bilateral
Duran, 2016 [24]	Observational N = 23	Retroaortic LRV (2 cases—8.7%).	Not mentioned.	Colombia	12 males 1 females	Left
Kumaresan, 2016 [25]	Observational N = 100	Retroaortic LRV (4 cases—4%). Multiple RV (19 cases—19%)	Not mentioned.	India	Not specified	Bilateral
Pandya, 2016 [26]	Observational N = 200	Circumaortic LRV (8 cases—4%). Retroaortic LRV (5 cases—2.5%). Double LRV (2 cases—1%) Double RRV (61 cases—30.5%), triple (5 cases—2.5%).	Not mentioned.	India	Not specified	Bilateral
Staśkiewicz, 2016 [27]	Observational N = 996	Circumaortic or retroaortic courses of the LRV in 99 cases (10%).	No significant difference was observed in the type of RV course between men and women (c2 = 1.22, *p* = 0.543).	Poland	481 males 515 females	Bilateral
Bouzouita et al., 2015 [28]	Observational N = 71	Retroaortic LRV (1 case—1.4%). Double LRV (3 cases—4.2%). Double RRV (5 cases—7%).	Not mentioned.	Tunisia	Not specified	Bilateral
Heidler, 2015 [29]	Observational N = 7929	Retroaortic LRV (61 cases—0.77%).	Not mentioned.	Austria	4781 males 3148 females	Left
Reginelli, 2015 [30]	Observational N = 921	Multiple RV (94 cases—10.2%). Retroaortic RV (219 cases—23.8%).	Not mentioned.	Italy	418 males 503 females	Bilateral
Resorlu, 2015 [31]	Observational N = 680	Retroaortic LRV (36 cases—5.4%). Circumaortic LRV (17 cases—2.5%). Multiple RV (46 cases—6.8%).	Hematuria was detected in 23.5% of patients with circumaortic LRV anomaly and 10.1% of patients without anomaly (*p* = 0.074). Hematuria was found in 21.7% of patients with multiple RV and 9.6% in those without the anomaly (*p* = 0.009).	Turkey	391 males 289 females	Left
Zhu, 2015 [32]	Observational N = 1452	Circumaortic LRV (31 cases—2.1%). Retroaortic LRV (30 cases—2.1%).	No statistically significant correlation found between left/right RV variations and sex (*p* > 0.05).	China	Not specified	Bilateral
Ballesteros, 2014 [33]	Observational N = 312	Circumaortic LRV (1 case—0.32%). Retroaortic LRV (2 cases—0.64%). Double LRV (1 case—0.32%). Double RRV (28 cases—8.9%), triple (5 cases—1.6%).	No significant difference between the presence of additional veins in men and women (*p* = 0.452) and extrahilar origin between RRV and LRV (*p* = 0.768).	Colombia	129 males 27 females	Bilateral
Boyaci, 2014 [34]	Observational N = 746	Circumaortic LRV (18 cases—2.4%). Retroaortic LRV (55 cases—7.4%).	No significant difference between the presence of variations [LRV (*p* = 0.801), RLRV (*p* = 0.551), CLRV (*p* = 0.823)] and sex.	Turkey	395 males 351 females	Left
Ferreira, 2014 [35]	Observational N = 1	Retroaortic LRV. Multiple RV.	29 to 65% of pyeloureteral obstructions were related to anomalies in the path of the vessels crossing the renal pelvis.	Colombia	1 males	Bilateral
Lavy et al., 2015 [36]	Observational N = 1	Multiple RV.	Not mentioned.	France	1 males	Bilateral
Rashid, 2014 [37]	Observational N = 100	Circumaortic LRV (3 cases). Retroaortic LRV (5 cases). Double RRV (16 cases—16%), triple (1 case—1%).	Not mentioned.	Iran	91 males 9 females	Bilateral
Șahin, 2014 [38]	Observational N = 2189	Circumaortic LRV (6 cases—0.3%). Retroaortic LRV (44 cases—2%).	Not mentioned.	Turkey	Not specified	Left
Dilli, 2013 [39]	Observational N = 1204	Circumaortic LRV (25 cases—2.1%). Retroaortic LRV (38 cases—3.2%).	Significant correlation between retroaortic LRV and gender (*p* = 0.036).	Turkey	642 males 562 females	Left
Eid et al., 2013 [40]	Observational N = 1	LRV origin: IVC. End: RRV.	Not mentioned.	Japan	1 male	Left
Poyraz, 2013 [41]	Observational N = 1000	Circumaortic LRV (3 cases—0.3%). Retroaortic LRV (65 cases—6.5%).	Diameters of the RRV and LRV were not significantly different (*p* = 0.1). Diameter of the anterior LRV was significantly greater than contralateral RV in its widest portion (*p* = 0.04).	Turkey	537 males 463 females	Left
Tao, 2013 [42]	Observational N = 378	Circumaortic LRV (8 cases—2.1%). Retroaortic LRV (7 cases—1.85%).	Not mentioned.	China	197 males 181 females	Bilateral
Apisarnthanarak, 2012 [43]	Observational N = 65	Circumaortic LRV (1 case—1.5%). Retroaortic LRV (1 case—1.5%). Double LRV (1 case—1.5%). Double RRV (19 cases—29.2%), triple (4 cases—6.2%). Right gonadal vein draining into the RRV (4 cases—6.2%).	Not mentioned.	Thailand	25 males 40 females	Bilateral
Atalar, 2012 [44]	Observational N = 739	Circumaortic LRV (6 cases—0.8%). Retroaortic LRV (17 cases—2.3%).	Not mentioned.	Turkey	425 males 314 females	Left
Bouali et al., 2012 [45]	Observational N = 120	Circumaortic LRV (6 cases—5%). Retroaortic LRV (5 cases—4.17%). Double RRV (22 cases—18.3%), triple (2 cases—1.7%).	Not mentioned.	France	79 males 41 females	Bilateral
Dilli, 2012 [46]	Observational N = 2644	Circumaortic LRV (27 cases—1.02%). Retroaortic LRV (44 cases—1.66%).	No statistically significant gender difference was found between LRV variations (*p* = 0.83).	Turkey	1204 males 1440 females	Left
Gupta, 2011 [47]	Observational N = 30	Circumaortic LRV (2 cases—6.6%). Retroaortic LRV (2 cases—6.6%). Double LRV (1 case—3.3%). Double RRV (1 case—3.3%).	Not mentioned.	India	Not specified	Bilateral
Yi et al., 2012 [48]	Observational N = 3	Circumaortic LRV (1 case). Retroaortic LRV (1 case).	Not mentioned.	Japan	1 male 2 females	Left
Costa et al., 2011 [49]	Observational N = 254	Circumaortic LRV (1 case). Retroaortic LRV (3 cases). Double LRV (4 cases—1.5%). Double RRV (24 cases—9.8%).	Dominance of venous variations on the right side, 12 times greater than on the left.	Brazil	Not specified	Bilateral
Kulkarni, 2011 [50]	Observational N = 102	Circumaortic RV (5 cases—5%). Retroaortic RV (1 case—1%). Multiple RV (7 cases—7%).	Not mentioned.	USA	Not specified	Not specified
Li et al., 2011 [51]	Observational N = 61	Anastomosis between the LRV and hemiazygos vein (51 cases—83.6%).	Significant differences when comparing operation time. Type 4 took longer (*p* < 0.05), type 5 shorter time (*p* < 0.05).	China	32 males 29 females	Left
Favaro et al., 2009 [52]	Observational N = 1	Venous communication between LRV and RRV. Kidneys without any relation to the IVC or common iliac veins.	Not mentioned.	Brazil	1 male	Bilateral
Turkvatan, 2009 [53]	Observational N = 59	Circumaortic LRV (2 cases—3.3%). Retroaortic LRV (3 cases—5%). Multiple RV (3 cases—5%).	Greater sensitivity and specificity of MDCT for renal venous anomalies.	Turkey	32 males 27 females	Bilateral
Kaneko et al., 2008 [54]	Observational N = 190	Multiple RV (25 cases—13%).	Not mentioned.	Japan	Not specified	Bilateral
Mir et al., 2008 [55]	Observational N = 1	Double RV bilaterally.	Not mentioned.	India	Not specified	Bilateral
Natsis, 2008 [56]	Observational N = 319	Circumaortic LRV (8 cases—2.5%).	Not mentioned.	Greece	173 males 146 females	Left
Tombul, 2008 [57]	Observational N = 60	Multiple RV (9 cases—15%)	Sensitivity of MDCT angiography for veins was 93%.	Turkey	Not specified	Bilateral
Yagci, 2008 [58]	Observational N = 783	Circumaortic LRV (15 cases—2%). Retroaortic LRV (23 cases—3%). Double retroaortic vein (4 cases—0.5%).	No statistically significant difference in ages.	Turkey	Not specified	Left
Holt, 2007 [59]	Observational N = 278	Retroaortic LRV (9 cases—3.2%).	Not mentioned.	UK	278 males	Left
Karaman, 2007 [60]	Observational N = 1856	Circumaortic LRV (17 cases—8.9%). Retroaortic LRV (68 cases—3.6%).	Not mentioned.	Turkey	Not specified	Left
Karazincir, 2007 [61]	Observational N = 277	Retroaortic LRV in patients (13 cases—9.3%) and controls (3 cases—2.2%).	Significantly higher incidence of varicocele in patients compared to controls (*p* = 0.018).	Turkey	Not specified	Left
Koc, 2007 [62]	Observational N = 1120	Circumaortic RV (62 cases—5.5%). Retroaortic RV (53 cases—4.7%). Multiple RV (210 cases—18.8%).	Not mentioned.	Turkey	588 males 532 females	Bilateral
Raman, 2007 [63]	Observational N = 126	Circumaortic LRV (10 cases—8%). Retroaortic LRV (3 cases—2%). Double LRV (10 cases—8%). Double RRV (28 cases—22%), triple (2 cases—2%).	Not mentioned.	USA	57 males 69 females	Bilateral
Namasivayam, 2006 [64]	Observational N = 48	Circumaortic LRV (1 case—2%). Retroaortic LRV (2 cases—4%). Double RRV (13 cases—27%), triple (1 case—2%).	Venous phase images showed significantly greater opacification of the left renal, gonadal, adrenal, and lumbar veins (*p* < 0.05).	USA	20 males 28 females	Bilateral
Arslan, 2005 [65]	Observational N = 1125	Retroaortic LRV (19 cases—1.68%).	Not mentioned.	Turkey	573 males 552 females	Left
Kawamoto, 2005 [66]	Observational N = 100	Circumaortic LRV (3 cases—3%). Retroaortic LRV (2 cases—2%). Small posterior branch that runs behind the aorta and drains into the IVC (6 cases—6%).	Not mentioned.	USA	Not specified	Left
Klemm, 2005 [67]	Observational N = 86	Retroaortic LRV (1 case).	Not mentioned.	Germany	86 females	Left
Janschek, 2004 [7]	Observational N = 119	Circumaortic LRV (7 cases—6%). Retroaortic LRV (3 cases—2.5%). Double LRV (7 cases—5.9%), triple (1 case—0.8%). Double RRV (21 cases—18%), triple (6 cases—5%).	Not mentioned.	Austria	58 males 61 females	Bilateral
Lin, 2004 [68]	Observational N = 170	Circumaortic LRV (16 cases—9.4%). Retroaortic LRV (2 cases—1.2%).	Groups 1 and 2 were similar in operation time (*p* = 0.90), blood loss (*p* = 0.45), warm ischemia time (*p* = 0.14), and hospital stay (*p* = 0.45).	USA	Not specified	Left
Yeh, 2004 [69]	Observational N = 186	Precaval RRV (9 cases—4.8%).	Not mentioned.	USA	Not specified	Right
Yesidag, 2004 [70]	Observational N = 1003	Circumaortic LRV (23 cases—3.2%). Retroaortic LRV (9 cases—0.9%).	Not mentioned.	Turkey	Not specified	Left
Senecail et al., 2003 [71]	Observational N = 2	Circumaortic LRV (1 case). Retroaortic LRV (1 case).	Not mentioned.	France	1 male 1 female	Left
Duques, 2002 [72]	Observational N = 34	Circumaortic LRV (1 case—2.9%). Double LRV (3 cases—8.9%).	Not mentioned.	Brazil	24 males 10 females	Left
Sebe et al., 2002 [73]	Observational N = 88	Left adrenal vein that drains into a double RV (4 cases—4.5%).	Not mentioned.	France	Not specified	Bilateral
Aljabri, 2001 [74]	Observational N = 1788	Circumaortic LRV (29 cases—1.62%). Retroaortic LRV (57 cases—3.18%).	Not mentioned.	Canada	929 males 859 females	Left
Shindo, 2000 [75]	Observational N = 166	Circumaortic LRV (1 case).	Not mentioned.	Japan	3 males 1 females	Left
Yoshinag, 2000 [76]	Observational N = 203	Retroaortic LRV (1 case).	Not mentioned.	Japan	Not specified	Left
Satyapal, 1999 [77]	Observational N = 1008	Circumaortic LRV (301 cases—30%). Retroaortic LRV (71 cases—7.1%). Additional RV (60 cases—6%).	Not mentioned.	South Africa	Not specified	Left
Pozniak, 1998 [78]	Observational N = 205	Circumaortic LRV (17 cases—8.3%). Retroaortic LRV (6 cases—2.9%).	Not mentioned.	USA	90 males 115 females	Bilateral
Trigaux, 1998 [79]	Observational N = 1014	Circumaortic LRV, (64 cases—6.3%). Retroaortic LRV (38 cases—3.7%).	The distance between the entrance to the IVC in case of a circumaortic variation and the distance in the case of retroaortic RV were not statistically different (*p* = 0.6).	Belgium	572 males 442 females	Left
Baptista-Silva et al., 1997 [80]	Observational N = 342	Circumaortic LRV (6 cases—1.75%). Retroaortic LRV (8 cases—2.3%). Double RRV (9 cases—2.63%), triple (3 cases—0.87%). Right gonadal vein draining into the RRV (1 case—0.3%).	Not mentioned.	Brazil	134 males 208 females	Bilateral
Hicks, 1995 [81]	Observational N = 108	Circumaortic LRV (11 cases—10%). Retroaortic LRV (2 cases—1.85%). Double LRV (5 cases—4.6%), triple (1 case—0.92%). Double RRV (18 cases—16.6%), triple (4 cases—3.7%).	No statistically significant difference between the 108 patients included and the 78 excluded regarding the indication for the procedure or demographic information such as sex, age, height, or weight. Renal venography was more sensitive in detecting both significant (*p* < 0.001) and insignificant (*p* < 0.001) abnormalities.	USA	51 males 57 females	Bilateral
Kaufman, 1995 [82]	Observational N = 150	Circumaortic LRV (8 cases—5%). Retroaortic LRV (10 cases—7%). Multiple RRV (12 cases—8%).	Not mentioned.	USA	Not specified	Bilateral
Satyapal, 1995 [83]	Observational N = 153	Double RRV (40 cases—26%), triple (5 cases—3.2%). Double LRV (4 cases—2.6%).	Not mentioned.	South Africa	131 males 22 females	Bilateral
Benedetti-Panici, 1994 [84]	Observational N = 309	Circumaortic RV (3 cases—0.97%).	Not mentioned.	Italy	309 females	Bilateral
Martinez-Almagro, 1992 [85]	Observational N = 116	Retroaortic LRV (6 cases—5%).	Not mentioned.	Spain	94 males 22 females	Left
Hoeltl, 1990 [3]	Observational N = 4520	Circumaortic LRV (4 cases—0.08%). Retroaortic LRV (29 cases—0.6%).	Not mentioned.	Austria	Not specified	Left
	Observational N = 354	Circumaortic LRV (2 cases—0.5%). Retroaortic LRV (4 cases—1.2%).				
	Observational N = 215	Circumaortic LRV (2 cases—0.9%). Retroaortic LRV (6 cases—2.8%).				
Monkhouse, 1986 [86]	Observational N = 57	Circumaortic LRV (2 cases—3.5%). Double RRV (1 case—1.7%). RRV drains into IVC lower than LRV (22 cases—38.5%). RRV drains into IVC upper than LRV (4 cases—7%).	Not mentioned.	UK	25 embalmed (9 males and 16 females); 32 fresh postmortem (8 males and 24 females)	Bilateral
Mayo, 1983 [87]	Observational N = 1140	Circumaortic LRV (1 case—0.08%).	Not mentioned.	Canada	Not specified	Left
Reed, 1982 [88]	Observational N = 433	Circumaortic LRV (19 cases—4.4%). Retroaortic LRV (8 cases—1.8%).	Not mentioned.	USA	Not specified	Left
Alexander, 1981 [89]	Observational N = 1200	Circumaortic LRV (3 cases—0.25%). Retroaortic LRV (1 case—0.08%).	Not mentioned.	USA	Not specified	Left
Beckmann, 1980 [90]	Observational N = 132	Circumaortic venous ring (8 cases—6.06%). Retroaortic LRV (1 case—0.75%). Double RRV (13 cases—9.84%), triple (3 cases—2.27%). Right gonadal vein draining into the RRV (4 cases—3%).	Not mentioned.	USA	Not specified	Bilateral
Kramer, 1980 [91]	Observational N = 193	Circumaortic RV (10 cases—5%).	Not mentioned.	South Africa	140 males 53 females	Left
Lien, 1977 [92]	Observational N = 100	Circumaortic LRV (10 cases—10%). Retroaortic LRV (2 cases—2%).	Not mentioned.	Norway	100 males	Left
Goswami, 1976 [93]	Observational N = 1	Double LRV.	Not mentioned.	USA	1 female	Left
Royster, 1974 [94]	Observational N = 159	Circumaortic LRV (1 case—0.6%). Retroaortic LRV (3 cases—1.8%), Double LRV (1 case—0.6%).	Not mentioned.	USA	Not specified	Left
Royster, 1974 [94]	Observational N = 228	Circumaortic LRV (1 case—0.43%). Retroaortic LRV (2 cases—0.8%).	Not mentioned.	USA	Not specified	Left
Davis, 1968 [95]	Observational N = 270	Circumaortic LRV (4 cases—1.5%). Retroaortic LRV (5 cases—1.8%).	Not mentioned.	USA	9 males	Left
Ross, 1961 [96]	Observational N = 34	Double RRV (7 cases—20.5%). Double LRV (1 case—3%).	Not mentioned.	Scotland	16 males 18 females	Bilateral
Reis, 1959 [97]	Observational N = 500	Circumaortic RV (30 cases—6%). Retroaortic LRV (12 cases—2.4%). Double LRV (4 cases—0.8%) Double RRV (51 cases—10.2%), triple (3 cases—0.6%).	Not mentioned.	USA	437 males 63 females	Bilateral

RV: renal vein; RRV: right renal vein; LRV: left renal vein; RLRV: retroaortic left renal vein; CLRV: circumaortic left renal vein; IVC: inferior vena cava.

**Table 2:** Prevalence studies included.

Author and Year	Total N	Prevalence	Multiple RV	RV Course (Circumaortic or Retroaortic)	RV Ramifications	Unusual Origin of RV
Pedrao, 2023 [13]	1	Multiple RV: 1	1	Not mentioned	Not mentioned	Not mentioned
Silva 2021 [14]	1	Multiple RV: 1 RV course: 1	1	1	Not mentioned	Not mentioned
Pradeep, 2020 [15]	188	Multiple RV: 12 RV course: 4	12	4	Not mentioned	Not mentioned
Salimy et al., 2020 [16]	1	Multiple RV: 1	1	Not mentioned	Not mentioned	Not mentioned
Fontana, 2018 [17]	1	Multiple RV: 1 RV course: 1	1	1	Not mentioned	Not mentioned
Tatarano et al., 2019 [18]	120	RV course: 7	Not mentioned	7	Not mentioned	Not mentioned
Dunnwald, 2019 [19]	1	Multiple RV: 1	1	Not mentioned	Not mentioned	Not mentioned
Shaheen, 2018 [6]	50	Multiple RV: 46	46	Not mentioned	Not mentioned	Not mentioned
Hassan, 2017 [20]	63	Multiple RV: 5	5	Not mentioned	Not mentioned	Not mentioned
Nambur, 2017 [21]	60	Multiple RV: 3 RV course: 7	3	7	Not mentioned	Not mentioned
Ayaz, 2016 [22]	222	RV course: 13	Not mentioned	13	Not mentioned	Not mentioned
Çınar, 2016 [23]	504	Multiple RV: 109 RV course: 47	109	47	Not mentioned	Not mentioned
Duran, 2016 [24]	23	RV course: 2	Not mentioned	2	Not mentioned	Not mentioned
Kumaresan, 2016 [25]	100	Multiple RV: 19 RV course: 4	19	4	Not mentioned	Not mentioned
Pandya, 2016 [26]	200	Multiple RV: 66 RV course: 13	66	13	Not mentioned	Not mentioned
Staśkiewicz, 2016 [27]	996	RV course: 99	Not mentioned	99	Not mentioned	Not mentioned
Bouzouita et al., 2015 [28]	71	Multiple RV: 8 RV course: 1	8	1	Not mentioned	Not mentioned
Heidler, 2015 [29]	7929	RV course: 61	Not mentioned	61	Not mentioned	Not mentioned
Mazengenya, 2015 [99]	1	Multiple RV: 1	1	Not mentioned	Not mentioned	Not mentioned
Reginelli, 2015 [30]	921	Multiple RV: 94 RV course: 219	94	219	Not mentioned	Not mentioned
Resorlu, 2015 [31]	680	Multiple RV: 46 RV course: 53	46	53	Not mentioned	Not mentioned
Ballesteros, 2014 [33]	312	Multiple RV: 34 RV course: 3	34	3	Not mentioned	Not mentioned
Boyaci, 2014 [34]	746	RV course: 73	Not mentioned	73	Not mentioned	Not mentioned
Ferreira, 2014 [35]	1	Multiple RV: 1 RV course: 1	1	1	Not mentioned	Not mentioned
Lavy et al., 2015 [36]	1	Multiple RV: 1	1	Not mentioned	Not mentioned	Not mentioned
Rashid, 2014 [37]	100	Multiple RV: 17 RV course: 8	17	8	Not mentioned	Not mentioned
Șahin, 2014 [38]	2189	RV course: 50	Not mentioned	50	Not mentioned	Not mentioned
Dilli, 2013 [39]	1204	RV course: 63	Not mentioned	63	Not mentioned	Not mentioned
Poyraz, 2013 [41]	1000	RV course: 68	Not mentioned	68	Not mentioned	Not mentioned
Tao, 2013 [42]	378	RV course: 15	Not mentioned	15	Not mentioned	Not mentioned
Apisarnthanarak, 2012 [43]	65	Multiple RV: 24 RV course: 2 RV ramifications: 4	24	2	4	Not mentioned
Atalar, 2012 [44]	739	RV course: 23	Not mentioned	23	Not mentioned	Not mentioned
Bouali et al., 2012 [45]	120	Multiple RV: 24. RV course: 11	24	11	Not mentioned	Not mentioned
Dilli, 2012 [46]	2644	RV course: 71	Not mentioned	71	Not mentioned	Not mentioned
Gupta, 2011 [47]	30	Multiple RV: 2 RV course: 4	2	4	Not mentioned	Not mentioned
Yi et al., 2012 [48]	3	RV course: 2	Not mentioned	2	Not mentioned	Not mentioned
Costa et al., 2011 [49]	254	Multiple RV: 28 RV course: 4	28	4	Not mentioned	Not mentioned
Kulkarni, 2011 [50]	102	Multiple RV: 7 RV course: 6	7	6	Not mentioned	Not mentioned
Li et al., 2011 [51]	61	RV ramification: 51	Not mentioned	Not mentioned	51	Not mentioned
Turkvatan, 2009 [53]	59	Multiple RV: 3 RV course: 5	3	5	Not mentioned	4
Kaneko et al., 2008 [54]	190	Multiple RV: 25	25	Not mentioned	Not mentioned	Not mentioned
Mir et al., 2008 [55]	1	Multiple RV: 1	1	Not mentioned	Not mentioned	Not mentioned
Natsis, 2008 [56]	319	RV course: 8	Not mentioned	8	Not mentioned	Not mentioned
Tombul, 2008 [57]	60	Multiple RV: 9	9	Not mentioned	Not mentioned	Not mentioned
Yagci, 2008 [58]	783	RV course: 42	Not mentioned	42	Not mentioned	Not mentioned
Holt, 2007 [59]	278	RV course: 9	Not mentioned	9	Not mentioned	Not mentioned
Karaman, 2007 [60]	1856	RV course: 85	Not mentioned	85	Not mentioned	89
Karazincir, 2007 [61]	277	RV course: 16	Not mentioned	16	Not mentioned	Not mentioned
Koc, 2007 [62]	1120	Multiple RV: 210 RV course: 115	210	115	Not mentioned	Not mentioned
Raman, 2007 [63]	126	Multiple RV: 40 RV course: 13	40	13	Not mentioned	Not mentioned
Namasivayam, 2006 [64]	48	Multiple RV: 14 RV course: 3	14	3	Not mentioned	Not mentioned
Arslan, 2005 [65]	1125	RV course: 19	Not mentioned	19	Not mentioned	Not mentioned
Kawamoto, 2005 [66]	100	RV course: 5 RV ramifications: 6	Not mentioned	5	6	Not mentioned
Klemm, 2005 [67]	86	RV course: 1	Not mentioned	1	Not mentioned	Not mentioned
Janschek, 2004 [7]	119	Multiple RV: 35 RV course: 10	35	10	Not mentioned	Not mentioned
Lin, 2004 [68]	170	RV course: 18	Not mentioned	16	Not mentioned	Not mentioned
Yeh, 2004 [69]	186	RV course: 9	Not mentioned	9	Not mentioned	Not mentioned
Yesidag, 2004 [70]	1003	RV course: 32	Not mentioned	32	Not mentioned	Not mentioned
Senecail et al., 2003 [71]	2	RV course: 2	Not mentioned	2	Not mentioned	Not mentioned
Duques, 2002 [72]	34	Multiple renal vein: 3 RV course: 1	3	1	Not mentioned	Not mentioned
Sebe et al., 2002 [73]	88	Multiple RV: 4 RV origin: 4	4	Not mentioned	Not mentioned	4
Aljabri, 2001 [74]	1788	RV course: 86	Not mentioned	86	Not mentioned	Not mentioned
Shindo, 2000 [75]	166	RV course: 1	Not mentioned	1	Not mentioned	Not mentioned
Yoshinag, 2000 [76]	203	RV course: 1	Not mentioned	1	Not mentioned	Not mentioned
Satyapal, 1999 [77]	1008	Multiple RV: 60 RV course: 372	60	372	Not mentioned	Not mentioned
Pozniak, 1998 [78]	205	RV course: 23	Not mentioned	23	Not mentioned	Not mentioned
Trigaux, 1998 [79]	1014	RV course: 102	Not mentioned	102	Not mentioned	Not mentioned
Baptista-Silva et al., 1997 [80]	342	Multiple RV: 12 RV course: 14 RV ramifications: 1	12	14	1	Not mentioned
Hicks, 1995 [81]	108	Multiple RV: 28 RV course: 13	28	13	Not mentioned	Not mentioned
Kaufman, 1995 [82]	150	Multiple RV: 12 RV course: 18	12	18	Not mentioned	Not mentioned
Satyapal, 1995 [83]	153	Multiple RV: 49	49	Not mentioned	Not mentioned	Not mentioned
Benedetti-Panici, 1994 [84]	309	RV course: 3	Not mentioned	3	Not mentioned	Not mentioned
Martinez-Almagro, 1992 [85]	116	RV course: 6	Not mentioned	6	Not mentioned	Not mentioned
Hoeltl, 1990 [3]	5089	RV course: 47	Not mentioned	47	Not mentioned	Not mentioned
Monkhouse, 1986 [86]	57	Multiple RV: 1 RV course: 2 RV origin: 26	1	2	Not mentioned	26
Mayo, 1983 [87]	1140	RV course: 1	Not mentioned	1	Not mentioned	Not mentioned
Reed, 1982 [88]	433	RV course: 27	Not mentioned	27	Not mentioned	Not mentioned
Alexander, 1981 [89]	1200	RV course: 4	Not mentioned	4	Not mentioned	Not mentioned
Beckmann, 1980 [90]	132	Multiple RV: 9 RV course: 16 RV ramifications: 4	9	16	4	Not mentioned
Kramer, 1980 [91]	193	RV course: 10	Not mentioned	10	Not mentioned	Not mentioned
Lien, 1977 [92]	100	RV course: 12	Not mentioned	12	Not mentioned	Not mentioned
Goswami, 1976 [93]	1	Multiple RV: 1	1	Not mentioned	Not mentioned	Not mentioned
Royster, 1974 [94]	159	Multiple RV: 2 RV course: 5	2	5	Not mentioned	Not mentioned
Royster, 1974 [94]	228	RV course: 3	Not mentioned	3	Not mentioned	Not mentioned
Davis, 1968 [95]	270	RV course: 9	Not mentioned	9	Not mentioned	Not mentioned
Ross, 1961 [96]	34	Multiple RV: 8	8	Not mentioned	Not mentioned	Not mentioned
Reis, 1959 [97]	500	Multiple RV: 58 RV course: 42	58	42	Not mentioned	Notmentioned

RV: renal vein.

**Table 3:** Risk of bias of included studies. Risk of bias assessment according to the JBI critical appraisal checklist.

Author	JBI Q1	JBI Q2	JBI Q3	JBI Q4	JBI Q5	JBI Q6	JBI Q7	JBI Q8	Bias Risk
Dunnwald et al., 2019 [19]									Low
Eid et al., 2013 [40]									Low
Favaro et al., 2009 [52]									Low
Ferreira et al., 2014 [35]									Low
Yi et al., 2012 [48]									Low
Goswami et al., 1976 [93]									Low
Mazengenya et al., 2015 [99]									Low
Mir et al., 2008 [55]									Low
Pedrao, 2023 [13]									Low
Salimy et al., 2020 [16]									Low
Senecail et al., 2003 [71]									Low
Silva, 2021 [14]									Low

**Table 4:** The Joanna Briggs Institute (JBI) critical appraisal checklist for case reports.

(1) Were patient’s demographic characteristics clearly described?	Yes	No	Unclear	Not applicable
(2) Was the patient’s history clearly described and presented as a timeline?	Yes	No	Unclear	Not applicable
(3) Was the current clinical condition of the patient on presentation clearly described?	Yes	No	Unclear	Not applicable
(4) Were diagnostic tests or assessment methods results clearly described?	Yes	No	Unclear	Not applicable
(5) Was the intervention(s) or treatment procedure(s) clearly described?	Yes	No	Unclear	Not applicable
(6) Was the postintervention clinical condition clearly described?	Yes	No	Unclear	Not applicable
(7) Were adverse events (harms) or unanticipated events identified and described?	Yes	No	Unclear	Not applicable
(8) Does the case report provide takeaway lessons?	Yes	No	Unclear	Not applicable

Overall appraisal: Include ▢ exclude ▢ seek further info ▢.

## Data Availability

Not applicable.
